# Autocrine and paracrine regulation of pituitary endocrine cells

**DOI:** 10.3389/fendo.2026.1812616

**Published:** 2026-05-28

**Authors:** Jianmei Yang, Lingyu Li, Xiangbo Xie, Changying Zhao, Chen Chen

**Affiliations:** 1 Department of Pediatric Endocrinology, Shandong Provincial Hospital Affiliated to Shandong First Medical University, Jinan, China; 2 Second Clinical Medical College, Shandong University of Traditional Chinese Medicine, Jinan, China; 3 Department of Endocrinology and Metabolism, The Affiliated Traditional Chinese Medical Hospital of Southwest Medical University, Luzhou, China; 4 Endocrinology and Metabolism, School of Biomedical Sciences (SBMS), Faculty of Health, Medicine, and Behaviour Sciences, The University of Queensland, St. Lucia, QLD, Australia

**Keywords:** cell culture, cell morphology, expression, pituitary gland, proteomics

## Abstract

The anterior pituitary gland is an important endocrine organ composed of a variety of key endocrine cells that regulate the functions of peripheral targeted organs, such as growth, development, behavior, lactation, reproduction and the stability of the internal environment and metabolism. This gland also regulates the functions of targeted peripheral endocrine organs, such as the thyroid, adrenal gland and gonads. In recent years, H&E and immunohistochemical staining, electron microscopic observation, pituitary endocrine cell line establishment and transplantation, flow cytometry, immunoblotting, qPCR, proteomics, ion channel and signal studies, among others, provided new knowledge of pituitary structure and function mostly in laboratory rodents under different physiological and pathophysiological conditions. Such investigations may contribute significantly to the research and treatment of human pituitary diseases. This review aims to provide a focused view of the rapid progress in new knowledge of the pituitary without repeating classical theories for endocrinologists and neuroendocrinologists.

## 1 Introduction

The regulation of hormone secretion from the anterior pituitary gland of mammals is a central issue in neuroendocrinology. In addition to the portal circulation transporting hypothalamic regulatory hormones to pituitary, the existence of various nerve fibers from hypothalamus in the anterior pituitary gland have also been identified (1). The presence, expression, and action of receptors, regulatory factors, and hormones in the pituitary gland, as well as the effects of newly discovered bioactive peptides and factors on hormone secretion in the anterior pituitary gland, have been frequently reported. Classically, the anterior pituitary gland contains mainly five homologous ectodermal primordia-specific endocrine cells in a highly regulated temporal, same hormone cells tightly networked, and spatially organized during embryonic development; namely, growth hormone-producing cells (GH cells), prolactin-producing cells (PRL cells), gonadotropin-producing cells (FSH/LH cells), adrenocorticotropic hormone-producing cells (ACTH cells), and thyroid-stimulating hormone-producing cells (TSH cells). The development of this gland has been studied extensively, and the specific mechanism of the precise temporal, spatial and hormone-dependent cell-networked patterns have been studied. At present, in the normal pituitary gland, cell transcription, posttranscriptional regulation, and the influence of transcription factors with different temporal characteristics work together to form the normal structure and function of the endocrine pituitary gland. The regulatory hormones and neurotransmitters in the hypothalamic nucleus, dorsolateral region, infundibulum, and near pituitary region regulate and control hormone synthesis and secretion and endocrine cell proliferation and differentiation (2, 3). Because rodents are commonly used experimental models, a detailed analysis of their pituitary gland encompassing morphology, cell biology, hormone synthesis and secretion, and the effects of diet and drugs are essential to elucidate fundamental pituitary physiology and regulation.

## 2 Structure biology of pituitary

### 2.1 Anatomy and structure of pituitary

**Figure 1:**
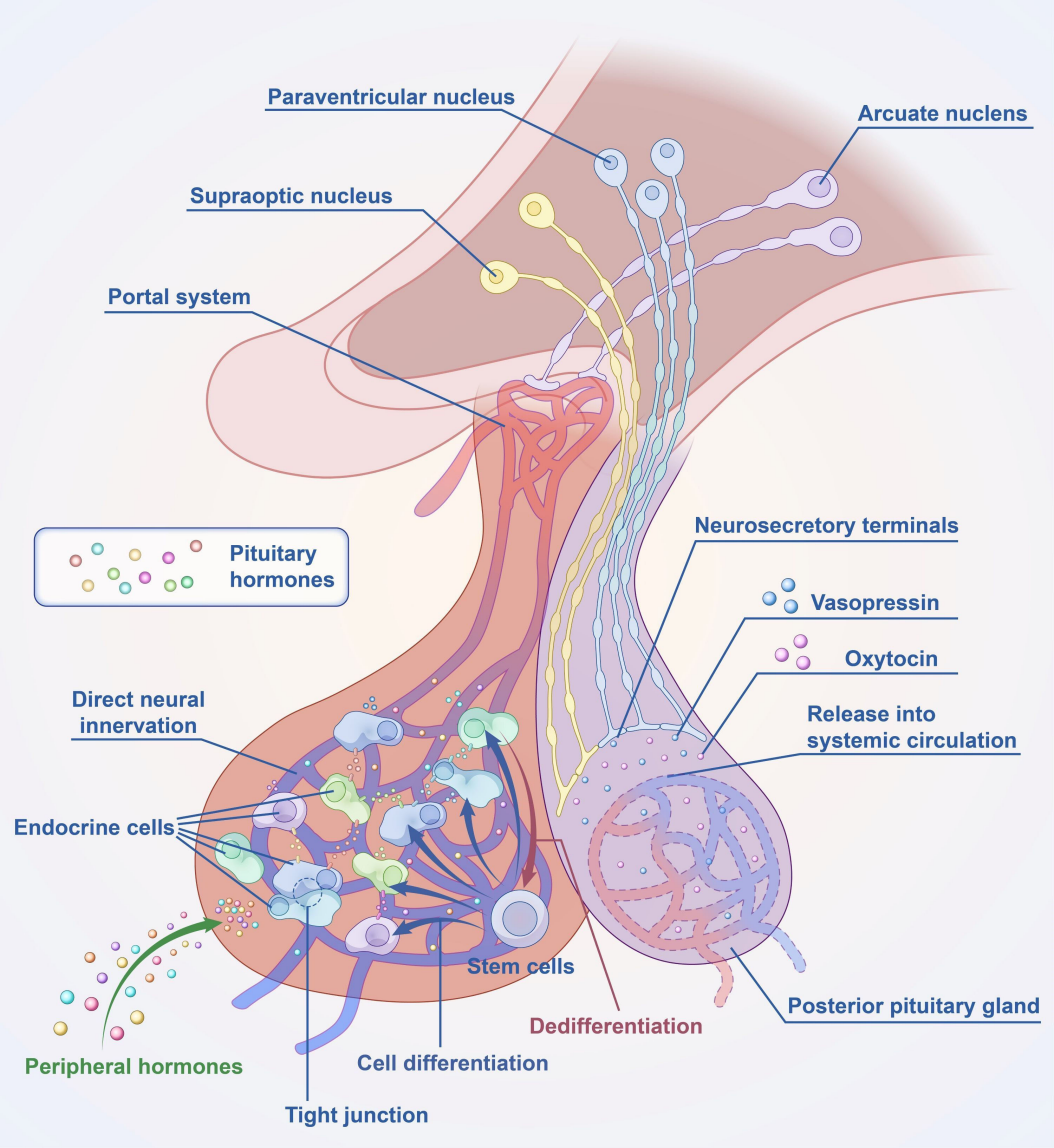
The anatomy of pituitary gland. The pituitary gland includes anterior pituitary (adenohypophysis) and Posterior Pituitary (Neurohypophysis). Adenohypophysis synthesizes and secretes a wide range of essential tropic hormones (ACTH, TSH, FSH, LH, GH, PRL). These hormones regulate growth, metabolism, reproduction, stress response, and the activity of other endocrine glands (thyroid, adrenals, gonads). Primarily controlled by releasing and inhibiting hormones produced by hypothalamic neurons. These hypothalamic hormones are transported to the anterior pituitary via a specialized hypothalamic-hypophyseal portal blood vessel system. Neurohypophysis does not synthesize hormones. Instead, it stores and releases two hormones produced by large neuronal cell bodies (magnocellular neurons) within specific hypothalamic nuclei (supraoptic and paraventricular nuclei): Oxytocin (involved in childbirth, milk ejection, bonding) and Vasopressin (ADH, Antidiuretic Hormone) (regulates water balance and blood pressure). Neurohypophysis hormone release is directly stimulated by neural impulses traveling down the axons of these hypothalamic neurons which terminate in the posterior lobe of pituitary gland.

The pituitary gland is a central and complex endocrine organ that secretes various peptide hormones and regulates the functions of peripheral endocrine glands and organs. Based on embryological development and histological characteristics, the pituitary gland is divided into two parts: the adenohypophysis or endocrine (anterior) pituitary gland and the neurohypophysis or neuronal (posterior) pituitary gland. The anterior pituitary gland is composed by dynamically differentiated endocrine cells from stem cells with tight junctions between same hormone cells and receiving mainly regulatory signals from portal circulation and direct neural innervation and peripheral hormones (**Figure 1**). The posterior gland receives neurosecretory terminals from paraventricular and supraoptic nucleus releasing vasopressin and oxytocin into systemic circulation (**Figure 1**). It was previously believed that the adenohypophysis originates from the primitive oral ectoderm, known as Rathke’s pouch, whereas the neurohypophysis arises from the neuroectoderm of the diencephalon, also referred to as the neurohypophysis bud or the infundibular pouch (4). Contrary to prevailing dogma, neural plate precursors in zebrafish (*her4.3^+^*) and mice (*Sox1^+^*) contribute to subsets of both neurohypophysis and adenohypophysis cells. The retinoic acid-degrading enzyme *Cyp26b1*, derived from pituitary cells, fine-tunes the differentiation of *prop1^+^* progenitor cells into hormone-producing cells (5). These findings challenge the notion that adenohypophysis cells are entirely derived from non-neuroectoderm and demonstrate that crosstalk between neurohypophysis and adenohypophysis cells influences pituitary cell differentiation (5). This discovery further challenges the traditional view that adenohypophysis cells arise exclusively from non-neuroectodermal lineages.

The pituitary gland is located at the base of the skull, in the pituitary fossa above the Sella turcica, occupying most of the pituitary fossa, and the remaining space is filled by the anterior cavernous sinus, cavernous sinus, and posterior cavernous sinus. The pituitary gland is suspended below the hypothalamus by the pituitary stalk. The pituitary gland is divided into the upper part of the sellar septum and the lower part of the sellar septum. The pituitary stalk is located in the upper part of the sellar septum, and the lower part of the sellar septum is mainly composed of endocrine tissue (6).

The pituitary gland consists of three major parts throughout early development: the distal, nodular, and middle parts (1). The distal part is the main component of the pituitary, where glandular cells gather into cords, clusters and networked same hormone containing cells, with abundant sinusoidal capillaries between the cells. Histological staining has shown that the distal part is composed of three types of cells. Among them, chromophobe cells account for approximately 50%, eosinophils account for approximately 40%, and basophils account for approximately 10%. Eosinophils are often distributed in the marginal zone, whereas chromophobes and basophils are mostly distributed in the central zone. Eosinophils are divided into two types based on the size of their secretory granules and immunohistochemical reactions: GH cells and PRL cells. Basophils have a large volume but vary in size, with diameters of approximately 15–25 microns and are mostly distributed in the central or cephalic portion of the distal region. The cells are circular, ovoid, or polygonal in shape. The cellular outlines are clear, and the cytoplasm contains basophilic granules of varying sizes, which are generally smaller than eosinophilic particles and less prone to staining with hematoxylin. The cytoplasm contains many mitochondria, and the Golgi complex is well developed. The basophil granules may be separated by gradient centrifugation and be divided into three cell types: thyrotropin cells or thyrotropes, gonadotropin cells or gonadotrophs, and adrenocorticotropin cells or corticotropes. There are many chromophobe cells distributed in clusters in the cell cords. Chromophobe cells are believed to be precursors of chromophil cells, which differentiate into eosinophils or basophils (2). The nodular area of the pituitary gland is separated from the funnel-shaped stalk by a thin layer of connective tissue, with a thickness of approximately 25–60 microns. As the pituitary portal vein needs to pass through this area, there are many blood vessels. The cells in the nodules are arranged longitudinally between blood vessels in a cord-like manner, are mostly cubic or columnar in shape, and have diameters of approximately 12–18 microns. The cells contain many small secretory granules, small lipid droplets, and many glycogen particles (3). The middle part of the gland is relatively narrow, accounting for approximately 2% of the pituitary gland in humans. It is composed of vesicular structures of varying sizes surrounded by some chromophobe cells and basophils. The basophils in the middle are mainly melanocyte-stimulating hormone-producing cells (MSH cells), which are large and polygonal in size. Their cytoplasm contains abundant mitochondria, a well-developed rough endoplasmic reticulum, and a Golgi complex, as well as many secretory granules with diameters of approximately 200–300 nm, and the electron density of the secretory granules varies (7). Far from being a random aggregation of cells, the pituitary gland possesses a highly organized spatial architecture that is essential for its function. This organization, which includes specialized microenvironments like the marginal cell layer and dense cell clusters, creates functional niches enriched with stem cells (e.g., SOX2-positive cells) that are critical for tissue homeostasis, plasticity, and regeneration. In rodent models, the distribution pattern of SOX2-positive cells closely resembles that in bovine pituitary, primarily localized in the MCL, dense clusters, and scattered individual cells within the parenchyma; demonstrating cross-species conservation of this architectural feature (8, 9). Additionally, the pituitary contains non-endocrine cells, such as folliculo-stellate (FS) cells, which mediate intercellular communication via calcium signaling and gap junctions to regulate endocrine cell activity (10). FS cells form a network within the pituitary, communicating with each other and other cell types, and are thought to be involved in regulating hormone secretion and potentially even stem cell renewal. The pituitary gland contains stem cell populations that are crucial for organ development, homeostasis maintenance, and injury repair. These stem cells are primarily SOX2-positive and may be classified into two distinct niches: Marginal cell layer niche (MCL-niche) and Parenchymal niche. In rodents, CD9/SOX2 double-positive cells in MCL-niche serve as the primary stem cell source, located adjacent to Rathke’s cleft and supporting pituitary cellular renewal. The SOX2-positive cells in Parenchymal niche form dense clusters that exhibit resistance to damage or physiological stimuli, providing a source of new cells (11, 12). In normal tissues, such as the gastric epithelium, Sox2 is expressed in stem and progenitor cells. However, genetic ablation studies reveal that Sox2 itself is dispensable for gastric stem cell and epithelial self-renewal and normal tissue regeneration (13). This indicates that while Sox2 is a marker of stemness, the maintenance of the stem cell pool in normal homeostasis can occur independently of Sox2 function, likely through compensatory mechanisms or other core transcriptional networks (13). It is worth noting that the role of Sox2 is not limited to gastric epithelium. In tissues of foregut origin, such as the esophageal and airway epithelium, Sox2 regulates epithelial differentiation in a dose-dependent manner. Its function is highly context-dependent, and different expression levels exert varying effects on epithelial development (14). The anatomy of pituitary gland was summarized in **Figure 1** with new development included.

### 2.2 Histology and immunohistochemistry

Immunohistochemistry uses specific antibodies (or antigens) to detect the distribution of antigens (or antibodies) in tissues and cells. Galanin (GAL) is a neurohormone or neurotransmitter and plays versatile physiological roles in the neuroendocrine axis, such as regulating food intake, insulin level and somatostatin release. A considerable number of galanin (GAL) immune-positive cells were observed in the anterior pituitary gland (15). Vesicular Glutamate Transporter 1 (VGLUT1) was detected in mouse anterior pituitary corticotropes (16). The presence of GAL-immunoreactive cells was confirmed in the anterior pituitary of rats (17). The presence of LH, FSH, and co-existence of LH–FSH gonadotrophs in the anterior pituitary gland was demonstrated using anti-β-LH and anti-β-FSH antibodies (18). Interferon was discovered in the anterior pituitary glands of rats with γ-immunoreactive positive fibers (19). These fibers surrounded the glandular cells and were closely connected to them. There were abundant blood vessels around the γ-immunoreactive positive fibers, with the fibers well in the vascular wall. In the anterior pituitary of rats, VGLUT2 expression exhibits a distinct pattern across glandular endocrine cells, with marked variability in its distribution. VGLUT2-positive signals are most prevalent in LH cells, demonstrating near-universal presence. Substantial expression is also observed in TSH cells, while FSH cells show moderate levels. In contrast, PRL, GH, and ACTH cells display minimal or negligible VGLUT2 expression, indicating limited functional involvement in these cell types (20). The function of such existence of VGLUT2 is still not clear.

Substance P (SP) and the immunoreactive nerve fibers were observed in the anterior pituitary glands of fetuses of different gestational ages (21). There were SP-immunopositive fibers in the anterior pituitary of fetuses, and their morphology and distribution changed along the development. The SP fibers were located mainly in the central area of the anterior lobe and the junction of the lateral area in contact with blood vessels and glandular tissue. Small amounts of calcitonin gene-related peptide (CGRP) (22), 5-hydroxytryptamine (5-HT) (23), somatostatin (SOM) (24), and substance P (SP) (23, 25, 26) positive nerve fibers were also detected in the anterior pituitary of rats. The SP-immunoreactive fibers are vasomotor regulators (25). The results of double labeling studies also suggested that these nerve fibers were closely related to ACTH, GH, TSH, and other endocrine cells (27, 28). Electron microscopy revealed that SP and CGRP fibers formed synapses with glandular endocrine cells (29, 30). GABA immune-positive terminals were distributed in the lateral and superficial parts of the anterior pituitary in rats (31). Moreover, the anterior pituitary of rats was revealed to be innervated by GABAergic nerve fibers, and GABAergic terminals directly regulated the secretion of anterior pituitary endocrine cells such as gonadotrophs, somatotrophs and thyrotropes.

Electric field stimulation of the pituitary gland for 10 minutes significantly increased the secretion levels of FSH and LH by probably stimulating nerve fibers in this gland (32). This stimulating effect was blocked by TTX, a selective sodium channel blocker, indicating the involvement of action potentials through the nerve fibers (32). PRL cells were detected by immunofluorescence staining of PRL-positive secretory granules (33). In the GH3 cell line (rat PRL- and GH-producing cells), PRL gene expression was observed (34). In human iPSC-derived pituitary cells, PRL-producing cells exhibit secretion reactivity similar to that in pituitary gland (33). GH cells may be detected by immunohistochemistry staining using GH antibodies, and reduced GH secretion is observed in cases of pituitary hypoplasia. GH cells in the anterior pituitary of rats were morphologically divided into three subtypes: type I (mature) contained large secretory granules with a diameter of approximately 350 nm; type II (intermediate) contained a mixture of granules of varying sizes; and type III (immature) contained small granules with a diameter of approximately 150 nm. GHRH-stimulated GH secretion is closely associated with glucocorticoids, and the three subtypes of GH cells exhibit differential sensitivity to GHRH (35). The three GH cell subtypes reflect differences in maturity and function directly related to glucocorticoid receptor activities, suggesting that glucocorticoid not only influences GH synthesis but also modulates secretory mechanisms. PRL cells display morphologically diverse secretory granules with larger diameters (700–900 nm) (36). Some studies have further noted that spherical granules in these endocrine cells range from 100 to 800 nm, reflecting substantial variability in granule size (37). Atypical cells, in contrast, exhibit smaller spherical granules (approximately 150–200 nm) (38). These cells represent transitional forms co-producing GH and PRL, with the granules including granules labeled exclusively for GH, granules labeled exclusively for PRL, and mixed granules labeled for both GH and PRL. Such dual-labeled cells possess the capacity for dual immunolabeling, particularly distinguishable using immunogold cytochemistry with 10 nm versus 20 nm gold particles, which allows clear differentiation between GH- and/or PRL-containing granules (39). The key characteristics distinguishing GH cells from PRL cells lie in differences in granule size and morphology. PRL cells exhibit significant variability in granule morphology, spanning multiple size ranges between typical and atypical subtypes.

### 2.3 Developmental biology of pituitary

The developmental progression of the pituitary gland involves the coordinated establishment of its structural organization and functional connectivity. Throughout this process, endocrine cells progressively adopt an organized spatial distribution and establish close associations with the developing vascular network, thereby creating the foundation for precise hormonal regulation.

The development of the pituitary is closely associated with the formation of its vascular architecture. The establishment of a dense capillary network enables efficient exchange between endocrine cells and the circulation. In particular, the hypothalamic-pituitary portal system plays a central role in delivering hypothalamic regulatory signals to the anterior pituitary, thereby linking structural development with endocrine function (40). In addition to vascular transport, regulatory factors may also diffuse through interstitial spaces surrounding portal vessels, suggesting the presence of complementary pathways for signal distribution within the developing gland (41). Pituitary polypeptide hormones are released into extracellular tissue fluids through exocytosis, absorbed by porous capillaries in the pituitary gland, and entering the bloodstream to exert their effects. The pituitary glands of cattle and rats have been studied by transmission electron microscopy (42). In the intercellular space, particles of similar size, morphology, and electron density have been observed in adjacent pituitary endocrine cells. The immunocolloidal gold labeling method have observed the endocrine granules of ACTH cells using transmission electron microscopy; these granules protrude outward from the cell membrane, and the immune gold-labeled secretory granules secreted outside the cell (43). The secretion mode of ACTH cells may involve exocrine gland apical release in addition to exocytosis. The pituitary of rats were carefully observed by electron microscopy (44). Overall, the development of the pituitary gland is characterized by the coordinated formation of spatial organization, vascular networks, and neuroendocrine connectivity. These processes collectively establish the functional framework required for precise hormonal regulation in the mature gland.

### 2.4 Cell differentiation

Cell differentiation is one of the key processes driving the maturation of pituitary function (45). During development and postnatal life, pituitary progenitor cells give rise to multiple hormone-producing endocrine cell types, including somatotrophs, lactotrophs, corticotrophs, thyrotrophs, and gonadotrophs (46). Transcriptional programs and intercellular signaling within the pituitary microenvironment (47). Throughout the entire process of pituitary development, gonadotropin-producing cells undergo a gradual differentiation process regulated by morphogenetic signals and transcription factors, which drive the transformation of progenitor cells into specific endocrine cell types (48). In addition to the generation of different endocrine cell types, pituitary cell differentiation is accompanied by the gradual establishment of functional properties, including hormone synthesis, storage, and regulated secretion. Recent studies using single-cell transcriptomic approaches have shown that differentiating pituitary cells progressively acquire gene expression profiles associated with endocrine function, reflecting their maturation into hormone-producing cells (49, 50). Recent evidence indicates that pituitary endocrine cell types are organized into distinct structural and functional networks rather than being randomly distributed, and their characteristic relationships with the vasculature may contribute to differences in hormone output (51). In addition, pituitary cell differentiation remains adaptable under physiological conditions. Changes in hormonal demand can influence the activity and composition of endocrine cell populations, suggesting that differentiated cells retain a degree of functional plasticity that contributes to maintaining endocrine homeostasis (52). Together, these findings indicate that pituitary cell differentiation involves not only the formation of specific endocrine cell types but also their functional maturation, spatial organization, and adaptive capacity.

### 2.5 Paracrine and FS cells

The anterior pituitary also contains a type of agranular chromophobic cell known as the follicular stellate cell (FS cell), which was first identified in 1953 (53). FS cells are in the endocrine pituitary of the anterior lobe, exhibit a stellate morphology, lack granules, and surround small follicle-like structures. FS cells interconnect via long cytoplasmic processes and account for approximately 5-10% of the total pituitary cell population (54). FS cells are situated within the lumens of small or pseudo-follicles distributed throughout the anterior pituitary. Their elongated cytoplasmic processes form a three-dimensional (3D) anatomical network that embeds pituitary endocrine cells. Through gap junctions, FS cells form networks with each other and with endocrine cells, mediating electrical signal transmission and metabolite exchange to maintain pituitary homeostasis (55). FS cells actively share both intra-pituitary and extra-pituitary neuronal signals, collectively providing an effective way to coordinate anterior pituitary function in response to physiological or pathophysiological conditions. Although FS cells do not produce any pituitary hormones, the characteristic long cytoplasmic processes in proximity to surrounding endocrine cells suggest a regulatory role through communication between cells (56, 57). The FS cells form an extensive and complex 3D network in pituitary gland, to perform scavenging activity through phagocytosis of degenerated cells, to regulate endocrine cells *via* releasing various growth factors and cytokines such as interleukin-6 (IL-6), leukemia inhibitory factor (LIF), basic fibroblast growth factor (bFGF), vascular endothelial growth factor (VEGF), and follistatin, and to evoke large-scale intercellular communication via the long processes and gap junctions (54, 57). In young rats, FS cells exhibit the highest mitotic index among pituitary cells, with the index decreasing along age. These FS cells in young rats may differentiate into endocrine cells, though such proliferation and differentiation are very rare in adult rats. Such adult proliferation may often be associated with tumor formation (58). Through IL-6, FS cells exert autocrine actions in the murine pituitary cell line (TtT/GF), promoting their own proliferation. IL-6 may also stimulate prolactin (PRL) and luteinizing hormone (LH) secretion, through a paracrine effect (59). The Hedgehog (Hh) signaling pathway is a critical cell-to-cell communication network that plays a vital role in embryonic development, tissue homeostasis, and regeneration in adults. In the context of the pituitary gland, ACTH homeostasis refers to the maintenance of a stable population of ACTH cells and the preservation of steady-state ACTH synthesis and basal secretion under physiological conditions. Studies indicated that Hh signaling was not critical for ACTH homeostasis but significantly involved in GH-producing somatotrophs and FS cells. Increased Hh signaling in FS cells stimulates GH production/release from somatotrophs via a paracrine mechanism, through releasing vasoactive intestinal peptide (VIP). Therefore, Hh signaling regulates GH production/secretion through FS cells as a paracrine hub within the VIP-GH regulatory circuit (60). FS-derived IL-6 plays a dual role in both normal and tumor pituitary environments: promoting tumor cell proliferation and inducing oncogene-induced senescence (OIS) to stabilize benign tumors. IL-6 may therefore initiate and maintain the senescence program in pituitary adenomas (61). FS cells consist of subpopulations with distinct gene expression profiles, some are pituitary stem cells characterized by expression of stem cell markers including SOX2, S100β (8), SOX9, PROP1 (62), GFRα2, and CD9 (63), according to the immunohistochemistry analysis (64, 65). Claudin-9 appears to be expressed in nearly all FS cells, forming tight junctions crucial for maintaining the integrity of the epithelial barrier. Such epithelial barrier function is critical for stem cell behavior and fate determination (66). In pituitary, IL-1β, IL-6, and TNFα are primarily secreted by FS cells (67). In fact, FS cells are a rich IL-6 source in pituitary, produced and secreted without exogenous stimulation (68, 69). In summary, FS cells are not merely supporting cells within the pituitary but perform a complex paracrine regulatory function. Through multiple signaling pathways, FS cells dynamically control pituitary hormone secretion, stem cell proliferation, tumour cell senescence and progression. Such senescence mechanism is not only a pathological byproduct in the pituitary tumor, but also essential component of tumor growth and homeostatic under self-regulation.

## 3 Molecular regulation of pituitary

### 3.1 Cell proliferation

As an important endocrine gland in the body, the pituitary secretes hormones that regulate almost all aspects of the physiological activity of the body. The proliferation and apoptosis of pituitary cells are often observed in disease models of animals or *in vitro* cultured pituitary cells (70, 71). Hypothalamic hormones, such as thyrotropin-releasing hormone (TRH), may promote the proliferation of anterior pituitary cells (72). Pituitary cells exhibit various signs of cell division or proliferation (73), such as proliferating cell nuclear antigen staining, aging signal-positive cells, and apoptotic cells in aging rat anterior pituitary. These findings indicate that there is a dynamic balance of cell proliferation, aging, and apoptosis in the pituitary of adult rats.

### 3.2 Apoptosis

Observations revealed that the nuclear membranes of pituitary gonadotropin-producing cells or gonadotrophs in thyroxine-treated rats were unclear or disappeared, mitochondrial ridges were broken or disappeared, and myeloid and vacuolar changes appeared (74). Moreover, the rough endoplasmic reticulum was swollen with degranulation by thyroxine treatment. Thyroxine has been shown to cause degenerative changes in the ultrastructure of rat pituitary cells. Excess thyroxine inhibits the activity of protein folding-associated enzymes by interfering with the proton gradient and redox environment within the rough endoplasmic reticulum (RER) (75, 76). This leads to the accumulation of unfolded proteins, triggering endoplasmic reticulum stress. This stress disrupts protein synthesis in the rough endoplasmic reticulum and inhibits nuclear transcriptional function. Consequently, it causes ultrastructural degeneration in pituitary gonadotroph cells, ultimately resulting in endocrine dysfunction.

In addition to endoplasmic reticulum stress, the mitochondrial pathway also plays a key role in the apoptosis of pituitary cells. Primary culture of anterior pituitary cells were conducted after added treatment of dexamethasone and medicine, here traditional Chinese medicine “Yougui” (77). After 3 days of cultivation, Annexin V-FITC/PI double staining, flow cytometry detection, and Cell Quest software analysis were used to study the anterior pituitary cell apoptosis. Rhodamine-123 staining and flow cytometry were used to quantitatively analyze the mitochondrial membrane potential of anterior pituitary cells. The decrease in the mitochondrial membrane potential in the dexamethasone plus Yougui group was significantly less than that in the dexamethasone alone group, indicating that the antiapoptotic effect of the Yougui pill was related to mitochondria function and involved in mitochondria-mediated cell apoptosis pathways.

### 3.3 Non-hormonal regulatory molecules

Beyond the regulation of pituitary cell proliferation and apoptosis, endocrine function is further modulated by a range of non-hormonal molecules. These molecules act as important regulators of hormone synthesis and secretion, contributing to the fine-tuning of pituitary activity under both physiological and pathological conditions.

Metabolic cues represent an important layer of non-hormonal regulation. A recent single-cell transcriptomic study demonstrated that high-fat diet exposure alters pituitary endocrine cell composition and gene expression profiles, including changes in somatotrope-related pathways, indicating that systemic metabolic conditions can directly influence pituitary cellular function (78). In addition, obesity has been shown to impair pituitary endocrine activity through disruption of intracellular stress-response pathways; specifically, defects in the IRE1α-XBP1 branch of the unfolded protein response in the pituitary contribute to endocrine dysfunction and systemic metabolic imbalance (79). Furthermore, metabolic hormone signaling also participates in pituitary regulation, as evidenced by studies showing that leptin receptor signaling in somatotropes is required for maintaining normal pituitary transcriptomic responses under high-fat diet conditions (80).

A single-cell transcriptomic study showed that systemic inflammatory challenges induce broad and cell-type-specific transcriptional responses in mouse pituitary hormone-producing cells, with corticotropes showing the strongest response (81). This study also demonstrates that pituitary hormone-producing cells upregulate the expression of chemokine genes under inflammatory conditions, supporting communication between endocrine and immune cells within the pituitary microenvironment. This suggests that the pituitary plays a multifaceted role in mediating the effects of inflammation on many aspects of the body’s physiology (81). In an ovine model, acute and prolonged lipopolysaccharide-induced inflammation altered the expression of pro-inflammatory cytokines and their receptors in the anterior pituitary, including IL-1β, IL-6 and TNF-α-related signaling components (69). The same study further reported that inflammatory stimulation was accompanied by changes in LHβ, GnRHR and FSHβ, indicating that local inflammatory responses in the anterior pituitary are associated with altered pituitary secretory activity (69).

Collectively, these findings indicate that non-hormonal regulatory factors are integral components of pituitary regulation, functioning alongside classical endocrine pathways to ensure precise control of hormone production and systemic homeostasis.

### 3.4 Protein molecules

**Figure 2:**
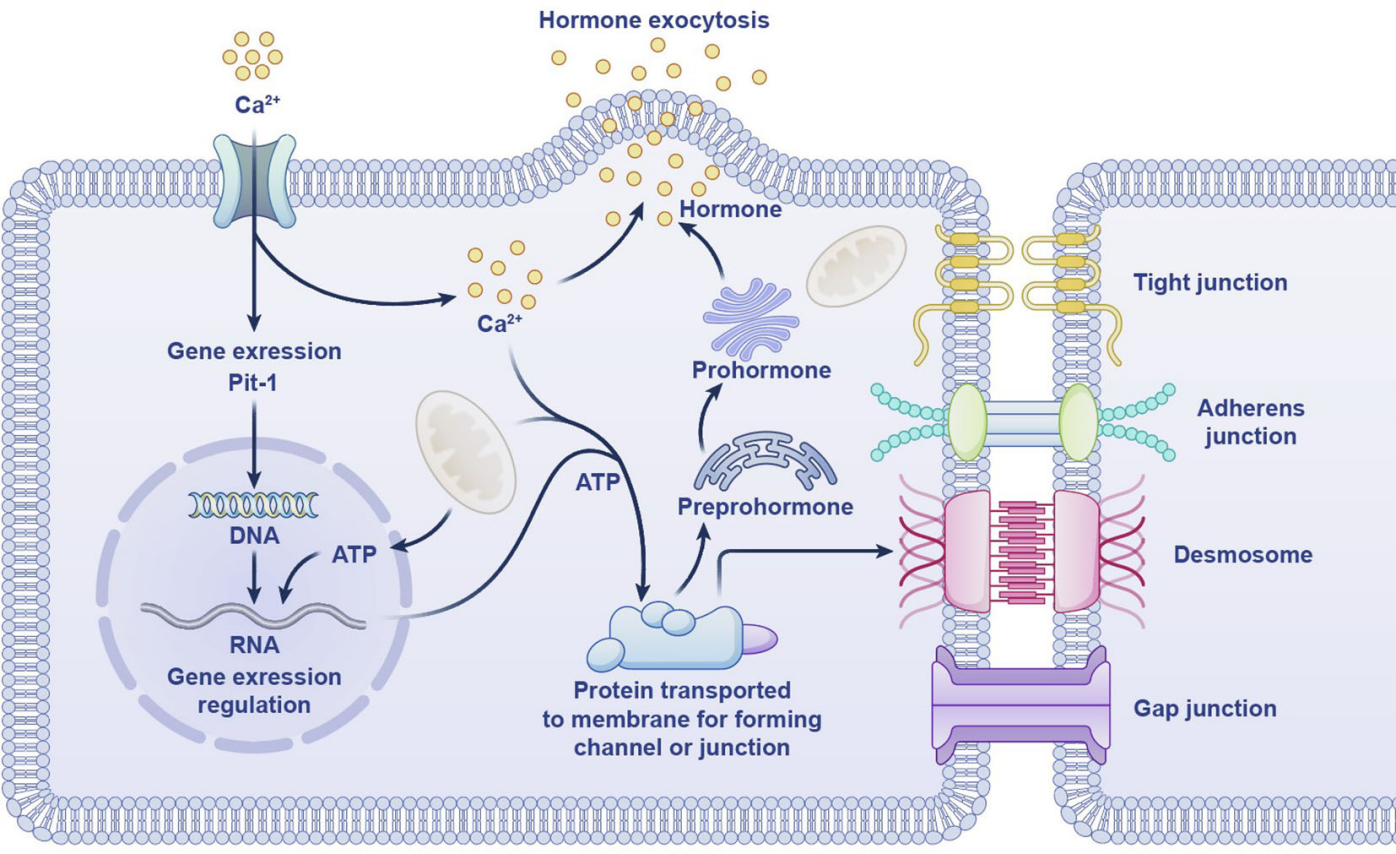
The connections between proteins, gene expression, and ion channels in pituitary endocrine cells. Hormone-secretory events activate signaling cascades that induce transcription factor activity, regulate gene expression, and integrated hormone secretion simultaneously through the network of same hormone cells. Network links include tight junctions, adherent junctions, desmosomes, and gap junctions between pituitary endocrine cells.

The secretion of pituitary gonadotropins in mammals has been studied focusing mainly on cellular morphology and the formation and release of dense secretory granules within cells (82, 83). The anterior pituitary controls various physiological processes by producing hormones from specific endocrine cells, and the levels of specific hormones reflect the capacity of pituitary to utilize pituitary plasticity in gene and protein expression across different hormone-producing cell populations. The proteins, LH, and glycoproteins in the purified vacuoles from pituitary, cerebral cortex, and liver tissues were analyzed by SDS-PAGE, Western blotting, and Con A/HRP (84). The results revealed the following: glycoproteins with different molecular weights were observed in the vacuoles of the pituitary, cortex, and liver tissues, but only the vacuoles of pituitary cells contained glycoprotein LH. Therefore, the vacuoles of pituitary cells may serve to store and release LH. Tandem mass tag (TMT) markers, HPLC classification, LC/MS, and bioinformatics analysis were used to investigate the effects of gonadotropin-releasing hormone (GnRH) on protein expression and phosphorylation in the rat endocrine pituitary (85). Exogenous GnRH therapy increased gonadotropin secretion without affecting the morphological structure of the adenohypophysis. One differentially expressed protein, GNA15, was increased by 1.5-fold as a significant molecule in the GnRH signaling pathway. Among the differentially expressed proteins (DEPs), multiple ribosomal proteins, which were crucial for reproduction and likely participated in gonadotropin synthesis, were identified (85). The phosphorylation levels of Protein Kinase C Alpha(PRKCA) were notably altered by GnRH, suggesting a potential role for PRKCA in GnRH signal transduction (86). TGFβ not only altered cellular motility but also significantly influenced the expression and interaction networks of various intracellular proteins (87). TGFβ treatment resulted in the upregulation of 51 proteins and the downregulation of 112 proteins. The affected proteins were primarily involved in biological processes such as actin cytoskeleton organization, cell adhesion, the extracellular matrix, and DNA replication. The pituitary gland exhibits intricate protein connections and molecular interactions regulating the pituitary cell functions. Recent advances in proteomic technologies have enabled comprehensive profiling of protein expression across pituitary subregions. A landmark study established the first lobe-specific proteomic atlas, identifying 4, 090 total proteins with 1, 446 demonstrating differential expression patterns between lobes - including the distinct regional distribution of pituitary hormones such as GH and TSH beta subunit (TSHβ) (88). Proteomic studies revealed that Prop1-expressing progenitor cells served as the common origin of endocrine cells, with their developmental trajectory being elucidated through Cre/LoxP mouse models (89). Furthermore, proteomic and phospho-proteomic analysis of rat endocrine pituitary following GnRH stimulation identified 6, 762 proteins and 15, 379 phosphorylation sites, including 28 upregulated and 53 downregulated proteins associated with hormonal secretion regulation (85). DEPs in porcine pituitaries were differentially expressed under environmental stress uncovering regulatory mechanisms of the hypothalamic-pituitary-adrenal (HPA) axis (90). Proteomic investigations of pituitary tumors revealed upregulated proteins including NOTCH3 and PTPRJ (91), which correlated with expression of autophagy-related proteins BECLIN1, and LC3 (92). Moreover, the proteomic signature of pituitary haemochromatosis indicated iron metabolism dysregulation specifically affecting anterior lobe function (93). Emerging multi-omics technologies such as spatial morpho-proteomics (INSIHGT), which integrates single-cell transcriptomics with proteomics, have been successfully applied to decipher the molecular networks of pituitary cells (94, 95). Proteomic technologies have significantly advanced the understanding of endocrine cell heterogeneity, differentiation regulation, and disease pathogenesis through high-resolution analysis of pituitary protein expression, post-translational modifications, and molecular interactions. In summary, most hypothalamic stimulatory hormones, such as GHRH (96), increase Ca^2+^ influx through Ca^2+^ channels, and such increase in intracellular Ca^2+^ in pituitary endocrine cells not only triggers the hormone secretion but also stimulates Pit-1 expression, and subsequent expression of hormones and membrane proteins to link differentiated same hormone cells (**Figure 2**).

### 3.5 Transcription factors

#### 3.5.1 Collaborative regulation and compensation mechanism of bHLH transcription factors in pituitary development

By analyzing triple-mutant mice for *Mash1*, *Math3*, and *Neurod*, the synergistic roles and mutual compensation mechanisms of these bHLH (basic helix-loop-helix) genes have been revealed in pituitary development. Morphologically, the adenohypophysis (anterior pituitary) of the triple mutants (*Mash1/Math3/Neurod*) resembles that of normal pituitaries, but the neurohypophysis (posterior pituitary) duct remains unclosed. The production levels of GH, FSH, and ACTH are significantly decreased, whereas PRL levels are increased. *Mash1* is involved in the differentiation of pituitary progenitor cells but insufficient alone to induce hormone-producing cell generation. *Mash1* is essential for the differentiation of ACTH and gonadotroph cells, in combination with *Math3* and *Neurod*. *Math3* is essential for somatotroph differentiation, in combination with *Mash1* and *Neurod*. *Mash1*, *Math3*, and *Neurod* cooperatively regulate the timing of progenitor cell differentiation during adenohypophysis development, which is essential for pituitary gland formation, and they functionally compensate for one another (97). *Neurod* likely acts in concert with other undetected bHLH factors, such as *Neurog2*, to ensure the integrity of pituitary development. These genes collaboratively control the timing of pituitary progenitor cell differentiation and are necessary for specifying subtypes of pituitary hormone-producing cells. Moreover, there is an evident compensation mechanism among these genes to ensure some degree of pituitary development even when certain genes are absent. These bHLH genes play crucial roles not only in pituitary development but also in maintaining the structural and functional integrity of the gland (97).

#### 3.5.2 Parasecretory regulation of ACTH-FGF1 signaling axis in pituitary structure and function maintenance

Fibroblast growth factor 1 (FGF1), which is produced as a paracrine signaling molecule by corticotropes, plays a critical role in determining pituitary structure and size. The intercellular communication mechanisms between different cell types were explored in the anterior pituitary, highlighting the critical role of corticotropes during the later stages of pituitary development (98). Blocking corticotrope differentiation led to hypoplasia of the pituitary and significantly impacted somatotrophs that were in direct contact with these cells. Genetic knockout experiments targeting the corticotrope-restricted transcription factor Tpit revealed that *Tpit*-deficient mice exhibited reduced growth hormone (GH), severely diminished secretory granules, and loss of cell polarity, ultimately resulting in systemic growth retardation (98). Single-cell transcriptomics revealed *Fgf1* as a corticotrope-specific, *Tpit*-dependent target gene responsible for mediating these cellular and phenotypic defects. Interestingly, the deletion of *Fgf1* was shown to phenocopy aspects of *Tpit* deletion, suggesting a functional link between these two factors in pituitary hypoplasia and growth disorders (98). The FGF1 produced by the corticotrope network is therefore a crucial paracrine signaling molecule involved in pituitary development for normal structure and size. The absence of *Fgf1* affects not only corticotropes themselves but also neighboring somatotrophs, indicating that FGF1 serves as a bridge in intercellular communication (98).

### 3.6 Ageing of pituitary

Senescent cells influence pituitary growth through the secretion of various factors (such as IL-6), forming the senescence-associated secretory phenotype (SASP) that exerts dual effects on pituitary development. For example, IL-6 not only participated in physiological pituitary growth but also mediated paracrine proliferative signals that impacted the tumor microenvironment (99). During pituitary injury repair, IL-6 is increased in young pituitary tissue, promoting stem cell proliferation and organoid formation. However, this regenerative capacity is significantly diminished in aged pituitary tissue (100), highlighting the crucial role of IL-6 in regulating pituitary stem cell activity and aging processes. Aging significantly affects pituitary hormone production, particularly prolactin (PRL) production. Research demonstrated that neuregulin-1 (Nrg1) delayed senescence of pituitary lactotroph cells and enhanced PRL secretion by activating the ErbB signaling pathway and increasing TRPM8 expression (101). Furthermore, melatonin improves pituitary cell function by enhancing the Nrg1/ErbB4 signaling pathway, thereby reducing the expression of senescence markers. Aging may also lead to pituitary fibrosis, as evidenced in pituitary tissue from elderly humans and aged rats. This fibrotic process may further compromise endocrine function, although the precise underlying mechanisms require additional investigation (102). The pituitary tumor transforming gene 1 (*PTTG1*) plays significant roles in cell replication, cycle regulation, and aging, influencing pituitary growth through protein interactions and transcriptional regulation (103). Other aging-related mechanisms, including oxidative stress, DNA damage, and telomere shortening, may also contribute to pituitary aging (104, 105).

## 4 Technical development in pituitary research

### 4.1 Single cell molecular biology

The application of single-cell RNA sequencing (scRNA-seq) in pituitary research has provided unprecedented insights into the cellular heterogeneity, developmental trajectories, and disease mechanisms of this complex organ. A representative study employing single-cell transcriptomic analysis of rat pituitary glands during sexual maturation identified 15 distinct cellular clusters and 106 differentially expressed genes, thereby uncovering the developmental dynamics of gonadotroph populations (48, 106). Pituitary stem cells (PSCs) exhibit an epithelial-mesenchymal hybrid phenotype and function as major signaling hubs (107). In adult rats, SOX2-positive stem cells display unique transcriptomic signatures (108), while human single-nucleus RNA-seq and ATAC-seq data further reveal dynamic alterations in stem cells during aging (109). Aged pituitaries show a significant downregulation of mRNAs related to protein translation machinery (110), and scRNA-seq of obese mouse pituitaries reveals dysregulation in cell metabolism and hormone secretion (106). The translational potential of these approaches is further exemplified by integrated single-cell and spatial transcriptomic analyses of pituitary neuroendocrine tumors (PitNETs), which have traced the trajectory of TPIT-lineage tumors and identified aggressive clusters marked by elevated p53-mediated proliferation, as well as SPP1+ tumor-associated macrophages that facilitate tumor invasion via the SPP1-ITGAV/ITGB1 signaling pathway (111).

The advent of scRNA-seq has transformed pituitary research by providing a potent instrument for accurate gene expression analysis at the single-cell level. This technology uncovers cellular diversity, classifies novel cell subtypes, tracks lineage decisions, and examines intercellular interactions, yielding invaluable perspectives for the advancement of innovative diagnostic and therapeutic approaches. Firstly, scRNA-seq facilitates high-resolution cell categorization and phenotyping. For instance, a comprehensive single-cell transcriptomic atlas of adult mouse pituitary has revealed previously unanticipated cellular complexity, including the identification of a Pou1f1-expressing multi-hormone cell population and the demonstration of sex-specific cell-type composition under normal homeostasis (112, 113). Secondly, scRNA-seq data enable the investigation of gene regulatory networks within pituitary cells. Studies have demonstrated dynamic shifts in cellular diversity and transcriptome profiles in response to physiologic stresses, underscoring the remarkable cellular plasticity of the pituitary gland (112, 113).The stem cell compartment has been particularly illuminated by these approaches: a detailed single-cell transcriptome atlas of male mouse pituitary across postnatal life has unveiled stem cell markers and niche regulatory factors, with findings functionally validated using pituitary stem cell organoids revealing roles for KLF5, AP-1, and EGF pathways in stem cell regulation (114). Furthermore, investigation of the neonatally maturing pituitary has decoded the activated stem cell phenotype, exposing a pronounced WNT pathway involvement in stem cell activation and demonstrating efficient regeneration capacity in the neonatal gland (78).

Although scRNA-seq itself lacks spatial context, it may be integrated with other methodologies to illuminate cell-to-cell interactions. Grasping how cells communicate within the pituitary tissue and their exact locations is crucial for understanding the collaborative and complex mechanisms that sustain tissue functionality and adapt pituitary gland to environmental fluctuations (49). Future advancements lie in multi-omics integration. Combining scRNA-seq with single-cell ATAC-seq, spatial transcriptomics, and methylation analyses may provide more comprehensive epigenetic regulatory networks. Furthermore, organoid models coupled with scRNA-seq offer novel tools for investigating pituitary development and pathogenesis (115, 116).

### 4.2 Pituitary cell lines

Pituitary cell lines provide a useful tool for neuroendocrine research on hormone release, transgenic modification, and cellular engineering. Current pituitary cell lines may maintain endocrine cell function with kinetic hormone secretion *in vitro*.

Pituitary transplantation may still be an effective alternative treatment for pituitary dysfunction. Pituitary cell culture and cryopreservation constitute the foundation of pituitary transplantation experiments. Studies have shown that cultured tissues or cells with reduced immunogenicity may survive well after xenotransplantation (117–120). There were reports of the effective transplantation of cultured pituitary cells in clinical practice for the treatment of pituitary dysfunction (121). Regarding the changes in the cellular composition and activity of cultured pituitary tissue cells, the anterior pituitary cell culture time is critical. The number of pituitary endocrine cells gradually decreases along culture time, accompanied by cell degeneration with the accumulation of endocrine granules. The purification of endocrine cells may increase the number of pituitary endocrine cells. The secretion of cultured pituitary cells is essentially consistent with changes in endocrine cell quantity, quality, cell structure, and vitality. Fibroblasts may be effectively removed during the primary culture period by inhibitors, and high-purity endocrine cells may be obtained after generations of passage using the same process, reaching a purity of over 80%, to eventually create pituitary cell lines. Current pituitary research primarily relies on animal models and tumor-derived cell lines, yet these models represent significant limitations, including substantial specie difference and loss of physiological functions, for instance, tumor-derived cell lines often exhibit impaired pattern of hormone secretion (122). Connexin 43 (Cx43) is the most ubiquitously expressed and extensively studied connexin in mammals. Connexins (Cxs) are critically involved in health, development, and homeostasis. They can disrupt tissue homeostasis by interfering with channel-forming capabilities, affecting signal transduction at the plasma membrane, cytoplasm, and even nucleus. Aberrant expression of connexins and gap junctions is frequently associated with tumorigenic phenotypes, where downregulation of Cx43 may contribute to tumorigenesis (123). The differentiation of human pluripotent stem cells (hPSCs) may develop into physiologically functional pituitary hormone-secreting cells, which demonstrate superior characteristics compared to tumor-derived cell lines (124). The CRISPR/Cas9 technology further facilitates the introduction of pathogenic mutations into hPSCs, enabling disease modeling and drug development (122). Pituitary SOX2-positive stem cells possess self-renewal capacity and multi-lineage differentiation potential, enabling the generation of all five hormone-secreting cell types (125). Validated stem cell models have been established in previous studies, providing a feasible pathway for functional regeneration (63). Studies using a total rat pituitary resection model demonstrated that transplanted pituitary tissue in the omentum survived and recovered function, as confirmed by morphological and functional assessments (126). Ectopic transplantation of hypothalamus-pituitary organoids improved physiological indicators in hypopituitary mice (127). Key challenges remain to be addressed, are ensuring long-term survival of transplanted cells, overcoming immune rejection, and achieving precise integration with the endogenous endocrine axis (128).

### 4.3 Pituitary organoids

Pituitary organoids may be established through two principal approaches: direct derivation from pituitary stem cells (129) or directed differentiation from human pluripotent stem cells (hPSCs), including both embryonic stem cells (ESCs) and induced pluripotent stem cells (iPSCs) (130). This organoid technology also enables the isolation and purification of pituitary cells for basic research and transplantation studies (131). The generation of hypothalamus-pituitary organoids (PHOs) from hPSCs successfully recapitulates *in vivo* microenvironments. Mouse transplantation studies have confirmed their functional restoration potential (124, 127). Notably, mouse pituitary-derived organoids demonstrated long-term expansion capacity while maintaining stem cell phenotypes, faithfully recapitulating the activated state of endogenous stem cells and their injury response mechanisms (132). Recent breakthroughs in human iPSC differentiation advanced organoid generation efficiencies that met or surpassed previous benchmarks through optimized culture conditions (133). The unique advantage of pituitary organoids in disease modeling enables precise pathological simulation. The successful modeling of congenital ACTH deficiency was achieved using CRISPR/Cas9-introduced pathogenic mutations in hiPSCs, with resultant organoids demonstrating patient-matched corticotrophin reduction phenotypes (134). The implementation of combined single-cell transcriptomics (scRNA-seq) and spatial transcriptomics (Stereo-seq) analyses has further revealed cellular heterogeneity, interaction networks, and spatial distributions within organoids (116). Compared to conventional models, these 3D structures better mimic the native pituitary microenvironment, overcoming the limitations of cell lines and addressing issues of lost cellular polarity and aberrant hormone secretion commonly observed in 2D culture systems (135). The development of pituitary organoids derived from embryonic stem cells (ESCs) or induced pluripotent stem cells (iPSCs) marks a significant advancement in pituitary research, as they effectively recapitulate pituitary development *in vitro* by mimicking the complex cellular differentiation, spatial organization, and hormonal functions observed *in vivo* (116, 136). Research has robustly demonstrated their value in disease modeling, where these organoids serve as three-dimensional experimental platforms to study pituitary disorders; for instance, optimized protocols enable high-efficiency differentiation and scRNA-Seq analyses reveal diverse cell clusters and intercellular interactions, including insights into genes like *SOX3* that regulate pituitary development, thereby facilitating investigations into congenital or acquired pituitary diseases (116, 137). Moreover, pituitary organoids offer an optimized alternative to traditional two-dimensional cell lines and pituitary transplantation therapies, as they generate purified, functional pituitary cells, such as ACTH cells, when transplanted into hypophysectomized animal models, successfully engraft and restore hormone levels, highlighting their potential for regenerative medicine applications (124). However, the technology still faces challenges including inter-organoid cellular composition heterogeneity and batch-to-batch variability, necessitating further optimization of culture protocols and characterization methodologies (138).

## 5 Conclusion

### 5.1 Pituitary cell types and heterogeneity

As a vital endocrine center of the body, the pituitary gland regulates various physiological functions by coordinating hormone secretion. Its anterior lobe consists of a variety of endocrine cells, including GH cells, PRL cells, ACTH cells, TSH cells, and FSH/LH cells cells, as well as non-endocrine cell populations such as FS cells and stem/progenitor cells. These cells are not randomly distributed or isolated from one another; rather, they are spatially organized and interconnected, forming an integrated regulatory network that collectively mediates the integration and transduction of signals from the hypothalamus and peripheral sources. With the advancement of research methods, our understanding of the pituitary’s complexity continues to deepen. Traditional immunohistochemistry and electron microscopy techniques remain indispensable for analyzing cellular morphology and ultrastructure, while rodent models and *in vitro* cell culture systems have laid the foundation for understanding the structure and function of the pituitary. Building on this foundation, emerging technologies including high-content imaging analysis combined with deep learning enable quantitative assessment of changes in subcellular structures, such as mitochondria, thereby linking structural changes to functional states. Most importantly, single-cell RNA sequencing has fundamentally transformed our understanding of the pituitary gland, revealing significant heterogeneity both within and between different classical endocrine cell lineages. These findings challenge the traditional “one cell type, one hormone” model and suggest that pituitary cells are more likely to exist along a continuum of transcriptional and functional states. Therefore, the heterogeneity of pituitary cells should be viewed as an integrated system determined by a combination of lineage differentiation, spatial organization, and cell-cell interactions.

### 5.2 Functional plasticity of pituitary cells

In addition to cellular heterogeneity, the pituitary gland exhibits significant functional plasticity, enabling it to adapt to ever-changing physiological and pathological demands. A growing body of research indicates that differentiated pituitary cells are not in a terminal state but can still modulate their gene expression, hormone secretion, and functional phenotype in response to signals from the internal and external environments.

This plasticity depends on a variety of interrelated mechanisms. Cell fate can be dynamically regulated in the pituitary microenvironment through multiple signaling pathways, with paracrine and autocrine actions playing particularly critical roles. FS cells play a central regulatory role in this process, acting as signaling hubs that mediate intercellular communication through the secretion of cytokines and growth factors, as well as via gap junction networks. Furthermore, pituitary stem/progenitor cells, particularly SOX2-positive stem cells, play a vital role in maintaining tissue homeostasis and facilitating regeneration, providing the cellular basis for the pituitary’s long-term adaptability.

In recent years, organoid models and lineage tracing techniques have provided essential tools for studying these processes. Findings from studies combining single-cell technologies indicate that pituitary plasticity is a key attribute linking cellular heterogeneity to functional adaptability. This is not only a critical mechanism for maintaining endocrine homeostasis but also plays a significant role in pathological processes such as pituitary tumorigenesis and endocrine dysfunction.
